# Editorial: Baby foods: Quality, safety, technology, and regulation

**DOI:** 10.3389/fnut.2022.1081287

**Published:** 2022-12-02

**Authors:** Adriana P. Arisseto, Florencia Cladera-Olivera, Tânia Gonçalves Albuquerque

**Affiliations:** ^1^Laboratory of Food Toxicology, School of Food Engineering, Department of Food Science and Nutrition, State University of Campinas (UNICAMP), Campinas, Brazil; ^2^Food Science and Technology Institute, Federal University of Rio Grande do Sul (UFRGS), Porto Alegre, Brazil; ^3^Department of Food and Nutrition, National Institute of Health Dr. Ricardo Jorge, Lisbon, Portugal

**Keywords:** infant formula, contaminants, nutrients, minerals, cereals

The full development, growth, and health of children depend on adequate nutrition in infancy and early childhood. Therefore, diet plays a central role in delivering key nutrients to meet an individual's physiological and nutritional needs, including components that cannot be synthesized by humans and must be obtained from foods, such as vitamins and minerals (1).

The World Health Organization (WHO) recommends that mothers worldwide initiate breastfeeding within the 1^st^ h of birth, and to exclusively breastfeed infants for the child's first 6 months. Breastmilk provides all the nutritional needs for the first number of months of life, as well as several benefits such as helping to protect children against various acute and chronic diseases. In addition to the introduction of complimentary food from the age of 6 months, the WHO recommends continuous breastfeeding up to 2 years old or beyond, since breastmilk continues to provide energy and important nutrients for the child's development (2).

However, breastfeeding may not be possible, sufficient, or prioritized for several reasons, including the mother's active work life or lack of breastfeeding experience (3). In these cases, infant formula is usually chosen to meet the nutritional demands of suckling infants (4). Infant formula is a product based on cows or other animals' milk, or a mixture thereof, which may also include other ingredients that have been proven to be suitable for infant feeding. The nutritional safety and adequacy of infant formula should be scientifically demonstrated to support the growth and development of infants (5).

In this sense, Alfaris et al. aimed to analyze and determine the chemical composition of a number of milk formulas available in the Saudi Arabian market. The authors verified that there were significant variations between milk formulas in relation to macronutrients, total energy, lactose, total solids, total non-fat solids, Ca, Fe, and Zn. The protein and mineral contents were within the recommended ranges; however, carbohydrate and fat contents were lower than the recommended ranges in the majority of investigated samples; this was highlighted as a concern by the authors.

Macrominerals (Ca, Mg, Na, K, and P) and trace minerals (Fe, Zn, Cu, Cr, Mo, Se, I, Co, and Mn) were investigated by Almeida et al. in commercial infant formulas marketed in Brazil. According to the obtained results, the highest macromineral concentrations were observed for Ca, K, P, and Na, and for the trace minerals Fe, Zn, Mn, and Cu. In general, samples met or exceeded Fe, Zn, Cu, Mo, and Se contents when compared with maximum limits established by international guidelines. Some macromineral and trace mineral contents presented values 20% below the values declared on labels; many samples also showed a nutrition content lower than the Adequate Intake (AI) for I, Na, Mn, K, and Mg, whereas Zn contents in some infant formulas were above the tolerable upper intake level (UL).

In addition to standard products, a varied range of infant formulas can be found on the market, including those containing added ingredients such as docosahexaenoic acid (DHA), prebiotics, probiotics, or even modified components such as reduced-lactose, hydrolyzed or soy proteins, and organic or non-GMO ingredients. These formulas claim to benefit the physical and mental development of babies as well as individuals with special conditions such as reflux and lactose intolerance (3). Gershman et al. examined the proportion of infant caregivers in the US who reported serving modified formula, and concluded that approximately half of participants serve it often, with sensitive and organic/non-GMO being the most common types of formula provided. According to the authors, the widespread provision of modified formula by infant caregivers raises concerns due to its higher cost and the lack of scientific evidence supporting the apparent benefits to babies.

Diversification in feeding usually begins at 6 months of age, using nutritionally adequate and safe complementary foods (2). These mainly include puréed vegetables, meat, or fruits, as well as juice, baby cereals, baby snacks, yogurt, soups, and others. Infants can be fed with home-prepared baby food or commercial products available on the market. Regardless the method of production (homemade or industrial), strict care must be taken in order to ensure high quality and safety of these products.

Some concerns may arise from excessive consumption of sugar, salt, and saturated fats, which have been linked to the development of several non-communicable diseases. As commercial infant cereals are among the first solid foods introduced to infants at the beginning of the complementary feeding period, and many infant cereals contain high levels of sugar and low percentages of whole grain, Sanchez-Siles et al. examined infants' overall acceptability of low-sugar complementary cereals. The authors observed that it is feasible to reduce the sugar content in infant cereals without sacrificing its sensory acceptability by infants and their parents.

The occurrence of toxic compounds in baby foods is also of major concern and needs to be addressed. Chemical contaminants can be naturally present in the raw materials used to produce these foods, can come from the environment (soil, water, or air), or can be formed during certain processes. The presence of carcinogenic polycyclic aromatic hydrocarbons (PAH) in baby food samples from Tehran and Iran was evaluated by Moazzen et al.. Low levels of PAH were found in the samples and the authors concluded that consumption of the examined foods does not pose a carcinogenic risk to health.

As intended, this Research Topic presented current and future challenges to ensure adequate infant food benefits and safety. There is a need for further studies in the area, including adequate monitoring of commercialized infant formulas. Considering that the health of adult populations is linked to malnutrition or obesity during childhood, aspects of baby food composition, quality, safety, and regulation require constant improvement.

## Author contributions

All authors listed have made a substantial, direct, and intellectual contribution to the work and approved it for publication.

## Conflict of interest

The authors declare that the research was conducted in the absence of any commercial or financial relationships that could be construed as a potential conflict of interest.

## Publisher's note

All claims expressed in this article are solely those of the authors and do not necessarily represent those of their affiliated organizations, or those of the publisher, the editors and the reviewers. Any product that may be evaluated in this article, or claim that may be made by its manufacturer, is not guaranteed or endorsed by the publisher.
